# Medical Opinion and Movement

**Published:** 1911-01-14

**Authors:** 


### Medical Opinion and Movement.

### Superheated Air for Prurigo.

In some cases of pruriginous dermatoses, such as kraurosis vulvæ, pruriginous eczema of the anus, etc., the irritation is so intense and causes so much distress to the patient that relief is sought by means of the cautery. Dr. Dreuw, of Berlin, finds that in such cases cauterisation may be substituted with success by simply allowing the current of heated air from the cauterising apparatus to play upon the part. Patients who have suffered for a long time from insomnia, in consequence of the pruritus, have obtained relief and enjoyed a good night's sleep after the first application. Dr. Dreuw generally makes one or two applications a day, each lasting about fifteen minutes. By adjusting the apparatus closer to or further from the body the temperature can be regulated according to the feelings of the patient. The author has treated successfully in this way several cases of scrotal eccema, kraurosis vulvæ, and anal pruritus. The method also shows good results in cases of acute moist eczema and in all sorts of skin ulcerations. It produces a drying effect and favours cicatrisation. Some years ago Dr. Audrey suggested the treatment of soft chancre in a similar way, and Dr. Ritter has obtained good results in the treatment of chilblains by superheated air.

### Dilution of the Blood in Fever.

Some interesting researches by Dr. Sandelowsky are reported in the *Deutsche Archiven für Klinische Medizin.* He has found that in all cases of fever there is a dilution of the blood, and that in the course of convalescence the blood assumes again its normal degree of concentration. In order to determine the cause of this phenomenon he produced an artificial pyrexia in rabbits, either by subjecting them to excessive heat for some time, and so causing a rise of temperature to 39°.5, and even 42°.5 C., or by cerebral puncture according to the method of d'Aronsohn and Sachs, or by injecting certain salts or dead cultures of the bacterium coli communis. As a result he finds that in all cases of pyrexia, however produced, there is the same dilution of the blood, whereas there are no parenchymatous changes in the tissues of the internal organs when the fever is produced by physical or chemical means such as occurs in fevers of an infective nature. So that this dilution of the blood is not dependent upon any such changes, resulting, for instance, in renal insufficiency. It appears, therefore, to be a biological reaction which occurs in a number of different conditions, having nothing in common but the pyrexia, and one is led to conclude that there is some causal connection between the two. Dr. Sandelowsky suggests that this hydration of the blood is a regulative phenomenon allowing the organisms to effect a greater loss of heat by the formation of sweat and the evaporation of water.

### A New Sign for Malaria.

In a contribution to *La Semaine Médicale* Dr. Ciro L. Urriola, physician to St. Thomas's Hospital, Panama, describes a means of determining the presence of malarial disease in a patient in whom failure to find the parasite in the blood by microscopic examination renders the diagnosis doubtful. As the author points out, the malarial parasite is not permanently present in the blood. It may disappear temporarily at the commencement of a febrile attack or under the influence of quinine, and it may be absent in certain pernicious forms with special localisation, and especially in all the latent forms and in malarial cachexia. On this account further diagnostic signs have been brought forward in the agglutinative power of malarial serum on normal blood and in the presence of urobilin in the urine. On various grounds, however, these two signs have been shown to be not altogether reliable or pathognomic. Dr. Urriola's sign consists in the presence of granules and masses of black pigment in the urine. At first the author sought for these pigment masses in the blood, but has now substituted examination of the urine. This latter method obviates the necessity of pricking the patient for the blood, and the pigment is much more abundant in the urine and therefore more easily found, and cannot be mistaken for anything else. According to the author these pigment masses can always be found in the urine of a malarial patient. The pigment occurs in fine granules and also in masses. Sometimes granules of a blue pigment are also found, either free or in hyalin plates, or in vesical epithelial cells. More rarely are seen masses and granules of pigment of an ochre colour. The author reports several interesting cases of an obscure nature in which the diagnosis of malarial origin was made by examination of the urine, and led to proper treatment and recovery of the patients.

### Whooping-Cough and the Motor-Car.

The favourable effects of a change of air in the later stages of whooping-cough are well known, and Dr. Manuel Rodriguez Portillo in *Clinica y Laboratorio* states that these results can be even bettered by ordering such patients to indulge in motor drives. He imposes certain conditions, however—namely, that a clear mild day be chosen for the excursion, a good level road selected, a speed of six and a half miles per hour never exceeded, and that the child be placed on the front seat of the car next to the chauffeur. He attributes the beneficial effects of this treatment to the increased depth of the pulmonary respiration thereby induced acting as a sort of sweeper-out of the respiratory passages, as well as to the tonic and stimulating action of the fresh air breathed. The appetite and blood-forming powers of the system are also increased.

### A Rare Complication of Otitis Media.

Dr. Rueda publishes in the *Revista de Medecina y Cirujia Practicas* a case which he cannot find paralleled in the literature. The patient, a man who had suffered for many years from middle ear disease with discharge, suddenly found that the latter had stopped. For a long time he was quite free from it, and no disease of the corresponding mastoid process could be discovered. It was supposed that he had undergone a spontaneous cure, until a few months ago he began to suffer from severe pain in the mastoid region, which no treatment could relieve and which finally necessitated an operation. At this the mastoid was found to be filled by a cystic mass, in contact with the lateral sinus, to the extent of 2 centimetres. During an attempt at enucleation this mass burst and gave issue to some very fœtid pus, which subsequent examination showed to be sterile. The author considers that this case is worthy of notice, in that it shows that the pus of otitis can collect and become sterile instead of increasing in virulence, when its free exit by the auditory canal is obstructed, and that in the process of becoming encysted a true fibrous membrane can be formed, which will allow of the purulent collection being tolerated by the surrounding tissues for a long period of time.

### The Value of Inoculation against Plague.

In an editorial on the value of inoculation against plague, *Indian Public Health* quotes figures given in the report of the Bombay Bacteriological Laboratory showing that of the total staff of 111 employed in killing rats, etc., all escape infection, though often bitten by plague-infected fleas. The staff is inoculated once a year. During the severe epidemic in Nagpur city some 22,347 persons were inoculated, and among them were 45 cases and 24 deaths from plague. Among 105,387 persons not inoculated there were 7,859 cases and 7,269 deaths, the percentages being 0.2 among inoculated and 7.4 among unprotected persons. In Bangalore, between October 1, 1907, and January 31, 1910, 76,743 persons were inoculated, while 25,384 remained unprotected. Deaths among the latter were 2,005 and among the former fifteen only. Other figures are given for Nasirabad and Bhusawal which correspond closely to those quoted above, and it is said that in the Punjab the incidence of plague amongst the unprotected is about thrice as great as amongst the inoculated, and that the chance of recovery amongst the latter is twice as great as amongst the former.

### Sea-Water Injections.

Cornelia de Lange, in a recent number of the *Nederlandsch Tydschrift voor Geneeskunde*, discusses, in an interesting article, the possibilities of Quinton's Plasma in diseases of children. In 1904 Rene Quinton, of Paris, recommended the subcutaneous injection of small doses of sea water in cases of tuberculosis, lymphadenitis, rickets, and scrofulous conditions generally. The sea water is now supplied in sterilised ampoules by a French firm, and is said to be particularly rich in salts of arsenic, cobalt, and barium. The technique of the injections offers nothing remarkable. The water is drawn up into a hypodermic syringe and injected subcutaneously, the injections giving rise to no pain or discomfort. Sometimes a marked reaction follows the first injection: the child's temperature rises a degree or more, the patient is restless, and there is a short period, which is quite transient, of irritability. The good effects of the sea water are manifest after the third or fourth injection, but observers vary considerably in their estimation of the value of such effects. The author finds that the injections appear to be beneficial in cases of long-standing eczematous conditions; but, on the whole, the conclusions given in this paper are discouraging, especially when compared with the somewhat enthusiastic championship of Quinton's remedy by Tixier and Simon, who highly vaunted the new method. Simon found the injections markedly curative in what he called "early stages of consumption"; but it is by no means certain that his child patients were consumptive, although they were all suffering from adenitis. De Lange has found some benefit resulting from the treatment in cases of gastric catarrh and general debility, but it is doubtful whether the results in such cases are legitimately to be ascribed to the injections.

### Constriction for Post-partum Hæmorrhage.

In a review of obstetrical progress contributed to the *American Journal of Obstetrics and Gynæcology*, Dr. G. L. Brodhead deals at some length with the method of treating dangerous post-partum hæmorrhage by constricting the aorta with strong rubber-tubing tied tightly round the waist. When the constrictor, which is an inch thick, has been properly applied, it compresses the aorta against the lumbar spine, and the pulse in the femoral arteries is obliterated. Hæmorrhage from the uterus then necessarily also stops, and it is said that the method has now been successfully used in several hundreds of cases. It is important to note that when the compression is to be removed it is advisable to raise the legs and apply a constricting bandage to each thigh, otherwise cardiac disturbances may occur. Momburg, who originated this plan in 1908, claims that no damage is done to the intestines or ureters, and that post-mortem findings have confirmed the harmlessness of the procedure. Kronig admits a few failures, ascribed to faulty technique, and lays stress on the control of the femoral pulse. Weber reports a long series of cases, nearly all successful, from Döderlein's wards. In gynæcological work it would seem from the reports that the method is not without danger. In very thin and debilitated subjects it has happened that direct strangulation of the intestine, with subsequent fatal peritonitis, may occur; and the presence of an ulcerated or chronically inflamed condition among any of the viscera would also naturally contra-indicate the use of "Momburg's belt." Another possible danger has been reported by Pagenstecher, who observed ischæmic paralysis of the distal part of the spinal cord.

### Free Acid in the Stomach.

A new and simple method of detecting the presence or absence of free hydrochloric acid in the stomach of a patient under suspicion of gastric carcinoma is described in the *Berlin. klin. Wochenschrift* by Dr. Fuld. The ordinary test meal is given to the patient after a night's fast. An hour later, instead of withdrawing the stomach-contents for testing, the patient is made to swallow some water containing bicarbonate of soda. If the viscus contains free hydrochloric acid, effervescence will then ensue, and can be distinctly heard if a stethoscope be applied to the abdominal walls over the region of the stomach. The absence of such sounds of effervescence suggests the possibility of absence of free acid, and the necessity for a stomach tube and a full analysis of the stomach-contents. Furthermore, when there is plenty of acid present, and when enough soda solution has been swallowed, the evolved carbon dioxide distends the stomach to such an extent as to render it tympanitic to percussion. To show this effect it is, of course, necessary to have percussed out very carefully the outlines of the stomach before giving the patient the alkali to swallow; and it is similarly advisable to auscultate the surface very carefully before carrying out the test, so that it may be certain that any effervescing sounds heard are really something which has originated *de novo* after the introduction of the bicarbonate of soda.

### The Internal Secretion of the Testis.

The hormone or internal secretion which is assumed to be manufactured in the testis and to account for the differentiated sexual characters of the male and female is as yet a purely hypothetical substance. Drs. Pappenheimer and Schwartz have conducted a long research to decide if possible by what parts of the testis this hormone is secreted; and they publish their conclusions in the *New York State Journal of Medicine*. The interstitial cells of the testis have always excited interest in this respect, and many observers have assigned to them the production of the internal secretion. They have been supposed by different observers to be of epithelial, endothelial, and connective tissue origin: the authors incline to the latter view. As to pathological processes but little is known. Hyperplasia so extreme as to lead to the formation of macroscopic tumours has several times been reported. In cryptorchid testicles their number is always markedly increased; and this feature, together with absence of normal spermatogenesis, is practically constant in ectopic testicles. The authors have examined sixty testicles, at all ages from infancy to seventy years. The interstitial cells are numerous about the fifth month of fœtal life, but diminish before birth. In childhood they are very scanty, but again become prominent at puberty. In adult life their number varies within wide limits, and no relation between these cells and spermatogenesis can be established.

Many observations seem to eliminate the spermatic epithelium from any part in the production of secondary sexual characteristics. Among the cases observed are two of interest—one of very marked hypoplasia of all sexual characters with hyperplasia of the interstitial cells of the testis, and one of normal sexual characters with marked testicular atrophy and hyperplasia of the interstitial cells. So that as far as these cases go it would seem very doubtful whether these cells can have any important *rôle* as hormone producers. It has often been noted that the subjects of the status lymphaticus present some anatomical features which may indicate some arrest of sexual development. In the present series there were eight cases of status lymphaticus, which had twice been the sole demonstrable cause of death. Seven of the eight subjects were adults, and active spermatogenesis was found in all of them. The amounts of interstitial cells and of the Sertoli (supporting) cells were also within normal limits; in fact, no morphological peculiarity could be observed. The authors do not confirm the observation of Kyrle as to the thickening of the membrana propria and the relative increase of supporting tissue in these cases. The too simple theory, they say, which ascribes the entire complicated series of changes attending the development of sex characters to the interstitial cells must be abandoned. They believe that other internal secretions besides that of the testis may be concerned, notably that of the hypophysis cerebri.

### The X-rays and Iron Metabolism.

In *Paris Médical* Dr. Lyon Caen summarises our present knowledge of the effects of *x*-rays on the metabolism of iron. Of late several accidents have been reported in cases where patients suffering from leukæmia were subjected to the action of the rays at frequent intervals or for lengthy periods of time. Oettinger, Fiessinger, and Sauphar have described a case in which a leukæmic patient developed an intense anæmia as the result of prolonged action at a distance of the rays. This they attribute to the apparition of a leucolysin, and not to the local and precocious effect of direct leucolysis. It is however possible that this anæmia is due to the action of the rays on iron metabolism. The spleen is an important reservoir for iron in the organism, and in both man and animals removal of the spleen increases the amount of iron excreted. Bayer has reported a case of myeloid leukæmia in which the excretion of iron was diminished. The rays induce an increase in the iron excreted, but this is only relative, for as compared with the normal the amount of iron excreted by a leukæmic subject is always low. This author has made a comparative study of the action of *x*-rays in two patients, of the same age and sex, one having a normal spleen, the other having had his removed. The result was an increase in the iron excreted by the former. From this he concludes that in certain leukæmias the capacity of the spleen to retain iron is diminished, and that the rays act on the spleen and increase the iron metabolism by causing it to throw out the iron it has stored up. In this way can be explained the stimulation given to iron absorption and its transformation into hæmoglobin by moderate doses of the rays, and also the anæmia produced by overdoses.

